# A tool to improve competence in the management of emergency patients by rural clinic health workers: a pilot assessment on the Thai-Myanmar border

**DOI:** 10.1186/s13031-015-0041-x

**Published:** 2015-04-13

**Authors:** Lilian Stanley, Thaw Htwe Min, Hla Hla Than, Marie Stolbrink, Kathryn McGregor, Cindy Chu, François H Nosten, Rose McGready

**Affiliations:** Shoklo Malaria Research Unit (SMRU), Mahidol-Oxford Tropical Medicine Research Unit, Faculty of Tropical Medicine, Mahidol University, PO Box 46, Mae Sot, Tak, 63110 Bangkok, Thailand; Mahidol-Oxford Tropical Medicine Research Unit (MORU), Faculty of Tropical Medicine, Mahidol University, Bangkok, Thailand; Centre for Tropical Medicine and Global Health, Nuffield Department of Clinical Medicine, University of Oxford, Churchill Hospital, Oxford, UK

**Keywords:** Medical emergencies, Training, Paramedical staff, Low resource setting, Knowledge, Confidence

## Abstract

**Background:**

Shoklo Malaria Research Unit has been providing health care in remote clinics on the Thai-Myanmar border to refugee and migrant populations since 1986 and 1995, respectively. Clinics are staffed by local health workers with a variety of training and experience. The need for a tool to improve the competence of local health workers in basic emergency assessment and management was recognised by medical faculty after observing the case mix seen at the clinic and reviewing the teaching programme that had been delivered in the past year (Jan-13 to March-14).

**Aims:**

To pilot the development and evaluation of a simple teaching tool to improve competence in the assessment and management of acutely unwell patients by local health workers that can be delivered onsite with minimal resources.

**Methods:**

A structured approach to common emergencies presenting to rural clinics and utilizing equipment available in the clinics was developed. A prospective repeated-measures observed structured clinical examination (OSCE) assessment design was used to score participants in their competence to assess and manage a scenario based ‘emergency patient’ at baseline, immediately post-course, and 8 weeks after the delivery of the teaching course. The assessment was conducted at 3 clinic sites and staff participation was voluntary. Participants filled out questionnaires on their confidence with different scenario based emergency patients.

**Results:**

All staff who underwent the baseline assessment failed to carry out the essential steps in initial emergency assessment and management of an unconscious patient scenario. Following delivery of the teaching session, all groups showed improved competence in both objective assessment and subjective confidence levels.

**Conclusions:**

Structured and practical teaching and learning with minimal theory in this resource limited setting had a positive short-term effect on the competence of individual staff to carry out an initial assessment and manage an acutely unwell patient. Health-worker confidence likewise improved. Workplace assessments are needed to determine if this type of skills training impacts upon mortality or near miss mortality patients at the clinic.

**Electronic supplementary material:**

The online version of this article (doi:10.1186/s13031-015-0041-x) contains supplementary material, which is available to authorized users.

## Introduction

Rural health clinics in low-resource settings around the world are predominantly staffed by paramedical staff with diverse and sometimes minimal levels of training. Training programmes can vary greatly between employing organizations whether they are national based government facilities or non-governmental organizations (NGOs). Little is known about learning and teaching in these environments as few standardized or published assessments exist. The majority of published literature focuses on teaching and training in acute trauma [1] and surgery [2-4], and in obstetric emergencies [5,6] but not on the management of acute medical emergencies in a rural low-resource setting. Low cost tools to improve human capacity in a context appropriate manner are required to improve initial assessment and potentially life-saving care, early in the clinical course of patients in rural areas of the developing world [7].

Shoklo Malaria Research Unit (SMRU) is an organisation which provides health care to the marginalised populations living on both sides of the Thai-Myanmar border in the Mae Sot area, Tak Province [8]. SMRU carries out dual activities of research combined with humanitarian work through five clinics which provide free health care to these vulnerable populations [9]. The majority of patients that attend for care come from Myanmar and out-patient and in-patient services are available. Patients requiring tertiary level care may be referred into the Thai hospital system.

The nature of the populations served by the clinics infers transportation and financial constraints which can lead to delayed presentation and a small percentage of patients can present with severe illness which require emergency management. Health workers at SMRU primary care clinics provide the first line of management for these medical emergencies and after hours support by doctors is usually only available by telephone. We set out to determine the existing level of emergency skills, and to pilot a relevant teaching programme for staff at the three busiest clinics to improve these skills.

A number of international ‘emergency’/acute care training days already exist and some of the health workers at SMRU have gone through Advanced Life Support in Obstetrics (ALSO®) training since 2008 originally developed in the United States of America. Language difficulties and the evidenced based, theory rich, content and the extended workshop design of such course presents both logistical constraints and management that is not possible or relevant to the low- resource setting in which the care is delivered. Competence in health literacy is low in patients [10] and although not formally measured in SMRU staff, difficulties with the theoretical component of ALSO® have been observed. An example of relevance in advanced life support is the use of intubation, defibrillation and medications such as amiodarone, which are simply not available in a remote and rural clinic.

The clinic staff regularly see and manage patients who are in shock secondary to sepsis or dehydration, but other medical emergencies are less common and the mainstay of management is to recognize the problem, stabilize the patient with oxygen, fluids and antibiotics if appropriate, and arrange transfer if needed. Based on this, we devised and piloted a short course that could be delivered to both medics and nurses. A medic in this setting has usually attended a training involving six to twelve months of theory and one year internship; and nurse training usually involves three months theory and three to six months of internship. Internships are normally supervised and involve on the job training. The focus of this training was recognition of danger signs and the early use of context appropriate and relevant interventions for typical and common emergency presentations.

## Methods

### Setting

SMRU is located in Tak Province on the Thai side of the border with Myanmar and a map of the clinic locations has been published previously [11]. The three busiest clinics were included for the piloting of the teaching tool: namely Mae La (MLA), Wang Pha (WPA) and Mawker Thai (MKT). MLA, 60 km north of Mae Sot, is the largest refugee camp in Thailand with an estimated population of 45,000 and the main health provider in the camp is Première Urgence – Aide Médicale Internationale. SMRU conducts maternal and neonatal health care in Maela with approximately 1,200 deliveries per year, a special care baby unit for neonates [12] and a small inpatient department where emergency care is required approximately 100 times per year. Services for migrants are estimated to serve a population of 200,000 and include WPA (30 km north of Mae Sot) and MKT (65 km south of Mae Sot) clinics. Combined these clinics admit an emergency cases 150 times per year, conduct approximately 1,200 deliveries per year and offer special care baby unit services, but manage a greater caseload of general out-patient and in-patient services. Previously patients of all ages with severe and hyperparasitaemic *P.falciparum* malaria were a daily occurrence and the staff were efficient in the acute resuscitation of these patients. The case-mix has changed dramatically over the past decade in the refugees [13] and past five years in the migrants [14]. There has been a significant reduction in malaria in the refugee and migrant communities with a relative increase in acute non-malaria presentations such as sepsis and obstetric complications [11]. The need for improved competence was recognised by medical faculty and in review of the teaching programme that had been delivered in the past year (Jan-13 to March-14).

### Developing the tool

As the aim was to improve competence in assessment and management, previous training tools involving clinical skills and past experiences at SMRU were discussed regarding their advantages and disadvantages [15,16]. Several of the physicians had attended various emergency courses in developed countries and certain aspects of these courses were discussed. Due to the limited depth and background education of the staff, theory components were kept to an absolute minimum. Preference was given to a structured approach to the emergency patient with equipment that was available in all clinics (limited options of emergency drugs, IV cannulas and only simple airway adjuncts). The risk that this approach would not be appropriate for every patient was recognized and balanced against the risk of losing staff attention in the details of the finer points of resuscitation. Drills based training has been observed to be well adapted to the background education of the staff and as this is based on a sequential and step-wise approach, which is also amenable to scoring, i.e. observed structured clinical examination (OSCE) [17], was chosen as the method to both train and assess.

### Piloting the tool

A prospective repeated measure objective assessment design was used alongside surveys carried out pre- and post- course to assess candidates’ confidence in managing different common presentations of emergency patients in this context.

The complete tool is described in Additional file 1. The outline of how we delivered the tool in this setting and the time frame of assessments is shown in Additional file 1, section 1.

### Assessment of competence

UK trained doctors carried out the assessment and training, each held current certificates in Advanced Life Support and had a minimum of 4 months experience working in Emergency Medicine departments. An initial ‘baseline’ assessment of medics and nurses (health-workers) knowledge and skills in emergency care was carried out prior to the training day. This was performed by giving each staff member a simple scenario of an acutely unwell patient:

*“A 45 year old female was admitted to IPD with pneumonia yesterday. Her breathing is fast and shallow. On admission she had a fever and was coughing green sputum. This morning when you go to take her vital signs you find her clammy and unresponsive”.*

The staff were then asked to talk through what they would do (real-time Myanmar and Karen interpreting was available). The interpreter, fluent in Karen, Myanmar and English, was a trained health care professional without formal teaching qualifications, employed by SMRU as a trainer, and had previously worked as a medic so the staff were comfortable with her.

Marking was carried out against a simplified ‘ABCDE’ structure where initial assessment of the acutely unwell patient should begin with assessment of the Airway (A) followed by Breathing (B), then Circulation (C) and signs of shock, Disability (D) including conscious level and basic neurological assessment, and finally Exposure (E) including a general examination and formulation of an ongoing plan. The order of the assessment relates to the urgency of the problem in each section e.g. ‘Breathing’, and any problems found in each section need to be addressed and management given before moving onto the next.

The use of this structure was agreed after consultation with the medical faculty. Additional file 1: section 3 shows the mark scheme used for the scenario assessments. The lines in grey are those actions that were ‘essential’ to be carried out in a basic emergency patient assessment and include assessing the airway, applying high flow oxygen and checking dextrose. Participants received a mark for each action regardless of when in the scenario it was carried out but the order of actions was recorded. Faculty agreed 14 ‘essential’ actions which we considered minimum requirement to pass the scenario.

Follow up assessments were carried out 1–2 weeks after the training using the same test scenario as the baseline assessments to enable direct comparison, with Burmese and Karen interpretation. No intervention e.g. correction or remedying of mistakes was undertaken in the assessments which were part of a pilot project aimed at teaching and improving these skills. Reinventing the scenarios would have invalidated comparisons between assessments. We liken this to any clinical teaching scenario where flaws in knowledge are identified but cannot all be addressed at once and time is taken to gain the appropriate knowledge, for example in all medical post-graduate exams and in ‘weekend’ life support courses.

Following the teaching, one site selected for remoteness (MKT) had fortnightly drill training as a group during the routine allotted teaching time. At each session a different scenario was practiced and the scenario was based on a real patient who had presented to the clinic (Additional file 1: section 7). Re-assessment was then carried out 8 weeks following the teaching day at all 3 sites (using the original scenario - Additional file 1 section 2), to examine whether there was any decline in knowledge or confidence. This time period was chosen in order to assess participants before they were exposed to other trainings or variables which could confound the outcomes.

All pre and post-training assessments were carried out by the same person and with the option of real-time Myanmar or Karen interpreting and the same interpreter used at each site. Participants who did not pass the post-test were allowed to retest but those results are not included in this data.

### Assessment of confidence

Data was anonymous for this section to encourage open feedback. Data was collected about age, role and previous training from each participant and they were asked to rank their confidence in dealing with 6 acutely unwell patient scenarios (Additional file 1: section 8).

### Teaching day

Nurses and medics at all 3 sites were invited to participate in the training, which was delivered over 4 hours (2 sessions, morning and afternoon) at each site and the same materials/structure and tutors were used for all 6 sessions). In order for work-flow at the sites to continue the staff organized themselves for duties and the number and availability of participants at each site varied. Not all staff could attend every session as the clinics required a skeleton staff to keep the clinic functioning on the teaching day. The days on which training was carried out based on availability of the interpreter and instructors. The training session was run twice at each site (morning and afternoon sessions on the same day) in order to allow the morning and afternoon shift staff to attend. Staff on night-duty were not called back especially for the assessment. No staff attended both the morning and afternoon sessions and no incentive to attend except the opportunity to learn was offered to participants.

The teaching was interactive, ‘hands-on’ and largely scenario based with each participant required to work through a different emergency scenario with help from colleagues (see Additional file 1: Appendix C day timetable). The learning process focused around instilling and practicing the ‘ABCDE’ approach to assessment and immediate management.

Participants filled in anonymous feedback forms on the quality of teaching received and were asked to re-rate their confidence levels in relation to the same scenarios.

### Statistical analysis

Analysis was conducted using SPSS version 20 for windows with comparison of proportions made using the Chi-squared test and the t-test and Mann–Whitney-U test for comparing parametric and non-parametric data, respectively. For staff who completed all three assessment points the Wilcoxon signed ranks test was used to compare the overall score and confidence scores.

### Ethical statement

The piloting of this tool was approved for use by the local Tak Province Community Advisory Board (T-CAB) who provide input on the ethics and conduct of studies, and of clinical and practice audits in this population [18].Written informed consent was obtained from participants. No risk was involved for the participants and participation was voluntary. All responses have been de-identified and names have been changed to ensure confidentiality.

## Results

### Participants and socio-demographics

The sampling frame consisted of 71 staff (45 nurses and 26 medics) working at the three different sites and overall, 24 participants went through the entire process from start to finish. The mean [range] age was 32 [19–57] years. There were only 11.3% (8/71) with recognized nursing diplomas from Myanmar and six of these participants had worked in Myanmar. Two-thirds (47/71) had received training from SMRU such as Basic Life Support in Obstetrics® or SMRU Nurse Training. Of those who completed the questionnaire, the majority 57% (37/65) had more than 5 years of work experience in their current job role with 15% (10/65) having worked for less than 1 year.

Selection of participants was reliant only on which medics and nurses gave consent and were able to attend the allotted days for pre-test, training and post-test. The number of staff available from each site to attend the baseline assessment, training, post-test assessment, and follow-up was variable (Table 1). The training day was well attended with the highest numbers of participants on this day (Table 1). The proportion of those who did two post-test assessments on the training day and again at the 8 week assessment was 79.7% (47/59) (Table 1).

**Table 1:** **Participant attendance per site at each step of the competence building exercise**

**SITE**	**Baseline assessment**	**Training**	**Post-test assessment**	**8 week follow-up assessment**	**All 4 steps**
	**N**	**N**	**n**	**n (%)**	**n**
MKT	15	26	22	14/22 (63.6%)	5
MLA	20	26	21	20/21 (95.2%)	10
WPA	14	19	16	13/16 (81.3%)	9
ALL SITES	49	71	59	47/59 (79.7%)	24

### Assessments

The results of the assessments were significantly better at the post-test assessment compared to baseline (Table 2) for median score and the proportion of participants who achieved a pass.

**Table 2:** **Assessments results**

	**Baseline**	**Post**	**8 week follow-up**	**P value**
	**N = 49**	**N = 59**	**N = 47**	
Score, median [range]	12 [5-21]	20 [12-24]	21 [14-25]	^**a**^ **P < 0.001**
				^b^P = 0.138
Passed, n (%)	0	42/59	31/47	^**a**^ **P < 0.001**
		(71.2)	(66.0)	^b^P = 0.674
Assessed Airway first	2/49	37/59	28/48	^**a**^ **P < 0.001**
	(4.1)	(62.7)	(58.3)	^b^P = 0.841
Assessed Airway in 1^st^ 5 steps	20/49	54/59	47/48	^**a**^ **P < 0.001**
	(40.8)	(91.5)	(97.9)	^b^P = 0.224
Give high flow oxygen in 1^st^ 5 steps	32/49	52/59	41/48	^**a**^ **P = 0.006**
	(65.3)	(88.1)	(85.4)	^b^P = 0.755
Reassess ABCDE after intervention	29/49	58/59	46/46	^**a**^ **P < 0.001**
	(59.2)	(98.3)	(100.0)	^b^P =1.000
Give correct fluid bolus	43/49	58/59	47/47	^**a**^ **P = 0.045**
	(87.8)	(98.3)	(100.0)	^b^P =1.000
Give correct amount fluid bolus	9/49	53/59	41/47	^**a**^ **P < 0.001**
	(18.4)	(89.8)	(87.2)	^b^P = 0.762
Check dextrose	29/49	59/59	45/47	^**a**^ **P < 0.001**
	(59.2)	(100.0)	(95.7)	^b^P = 0.194
**Attended all 3 assessments**	N = 24	N = 24	N = 24	
Score, median [range]	12 [5-21]	21 [17–24]	22 [14–25]	^**a,c**^ **P < 0.001**
				^b,c^P = 0.245
Passed, n (%)	0	18 (75.0)	18 (75.0)	^**a**^ **P < 0.001**
				^b^P = 1.000
Assessed Airway first	2 (8.3)	15 (62.5)	12 (50.0)	^**a**^ **P < 0.001**
				^b^P = 0.561
Assessed Airway in 1^st^ 5 steps	13 (54.2)	23 (95.8)	24 (100.0)	^**a**^ **P = 0.002**
				^b^P = 1.000
Give high flow oxygen in 1^st^ 5 steps	13 (54.2)	23 (95.8)	21 (87.5)	^**a**^ **P = 0.002**
				^b^P = 0.609
Reassess ABCDE after intervention	10 (41.7)	24 (100.0)	24 (100.0)	^a^ **P < 0.001**
				^b^P n.a.
Give fluid bolus	12 (50.0)	23 (95.8)	24 (100.0)	^**a**^ **P = 0.001**
				^b^P =1.000
Give correct amount fluid bolus	4 (16.7)	22 (91.7)	23 (95.8)	^a^ **P < 0.001**
				^b^P =1.000
Check dextrose	15 (62.5)	24 (100.0)	24 (100.0)	^**a**^ **P = 0.002**
				^b^P n.a.

^a^P value comparing Post vs baseline assessments.

^b^P value comparing Follow-up vs Post assessments.

^c^P value paired t-test Wilcoxon signed ranks test.

There was no significant difference in median score or the proportion of participants who passed at the 8 week assessment compared to post-test, although the majority of specific task comparisons showed a decreased proportion of actions were carried out (Table 2).

The number of staff who participated through all four components of the competence training was 40% (24/60) and their results have also been summarized (Table 2) with very similar outcomes to those who were unable to attend all assessments.

At baseline, the median score was low at 12 (Table 2) out of a maximum of 25 with poor scores across all three sites (Table 3): MKT 12, MLA 13 and WPA 10.5; and, likewise low pass rates. Post-test assessment results were much improved with a median score of 20 and a 71.2% (42/59) pass rate overall.

**Table 3:** **Differences between the sites at each assessment including confidence score**

		**Baseline**	**Post**	**8 week follow-up**	**P value**
MKT	N	15	22	14	
	Score, median [range]	12 [8–16]	21 [15–23]	22 [19–23]	^**a**^ **P < 0.001**
					^b^P = 0.204
	Passed, n(%)	0	16 (72.7)	13 (92.9)	^**a**^ **P < 0.001**
					^b^P = 0.209
	Confidence score	2 [1–4]	3 [3–4]	na	^a^P = 0.158
MLA	N	20	21	20	
	Score, median [range]	13 [7–21]	21[17–24]	21 [14–25]	^**a**^ **P < 0.001**
					^b^P = 0.916
	Passed, n(%)	0	17 (81.0)	9 (45.0)	^a^P < 0.001
					^b^ **P = 0.025**
	Confidence score	3 [1–4]	3 [2–4]	Na	^**a**^ **P < 0.001**
WPA	N	14	16	13	
	Score, median [range]	10.5 [5–15]	20 [12–23]	21 [15–23]	^**a**^ **P < 0.001**
					^b^P = 0.063
	Passed, n(%)	0	9 (56.2)	9 (69.2)	^**a**^ **P = 0.001**
					^b^P = 0.702
	Confidence score	2 [1–3]	3 [1–4]	Na	^**a**^ **P = 0.002**

^a^P value comparing Post vs baseline assessments.

^b^P value comparing Follow-up vs Post assessments.

While the majority of participants at baseline assessment gave a fluid bolus (87.8%, 43/49), less than 1 in 5 participants (18.4%, 9/49) gave the recommended volume of 250-500 ml volume of normal saline required for a shocked adult patient. There was a wide and potentially dangerous discrepancy in the volumes being given from 10 ml per kg to 3 litres.

At the post-test assessment almost all participants (98.3%, 58/59) gave a fluid bolus and a large majority (53/59 89.8%) gave the correct fluid and volume.

There was a general lack of recognition amongst staff that the scenario was an ‘emergency’ or ‘ABCDE’ patient. Some participants were able to quote ABCDE but then unable to qualify with what they stood for or how to assess each area e.g. A is for airway.

A significant rise in confidence was observed at two of the three sites between baseline and post assessments as shown in Table 3. A significant difference in confidence scores was observed for medics and nurses on all scenarios except the unwell pregnant woman, where the medics did not report increased levels of confidence (Table 4).

**Table 4:** **Confidence levels of nurses and medics**

	**Nurse**			**Medic**		
	**Baseline**	**Post**	**P value**	**Baseline**	**Post**	**P value**
	**N = 30**	**N = 45**		**N = 19**	**N = 27**	
**Anaphylaxis**	2 [1–3]	3 [1–4]	**0.003**	3 [1–4]	3 [2–4]	**0.003**
**Acute abdo**	2 [1–3]	3 [1–4]	**0.001**	3 [1–4]	3 [3–4]	**0.002**
**Acute SOB**	2 [1–3]	3 [1–4]	**<0.001**	3 [1–4]	3 [2–4]	**0.004**
**Sepsis**	2 [1–3]	3 [1–4]	**<0.001**	3 [1–4]	3 [2–4]	**0.010**
**Unwell PW**	2 [1–3]	3 [1–4]	**<0.001**	3 [1–4]	3 [1–4]	0.288
**Unconscious**	2 [1–3]	3 [1–4]	**<0.001**	3 [1–4]	3 [2–4]	**0.021**

Where 1 = very unsure, 2 = a bit unsure, 3 = a bit confident and 4 = very confident.

## Discussion

The level emergency skills found at all 3 sites at baseline was surprisingly low considering that staff at these sites see emergency patients on a regular basis. This demonstrates that the traditional “exposure” and “on-the-job” training is not enough to equip staff with the necessary emergency skills and therefore practical, focused training is vital. It could be argued that the poor baseline scores were because the staff were being tested in an approach which was unfamiliar. However ‘ABCDE’ is not a new concept to staff (it is referred to in the SMRU clinical guidelines) and although a minority of participants could recognize that an unconscious patient (test scenario) was someone who required “ABC”, very few could discuss how to assess the airway or what ‘A’ stood for.

It seems that part of the success of the teaching was because it was not based on learning facts. We taught a systematic approach with comparatively little focus on specific medical conditions, in order to equip staff with an approach they could apply to all emergency patients. We covered a number of common acute scenarios during our teaching but there was no didactic teaching involved and all sessions were interactive. As explained earlier our rationale for this style of teaching was based on faculty experience both at SMRU [15,16] and elsewhere, along with literature describing other similar projects successfully delivered in this style [19].

Table 1 shows that despite an overall reduction in scores by 6 weeks, the pass rates remain significantly higher compared with the pre-course rates, as would be expected from similar teaching projects such as the essential Emergency Triage Assessment and Treatment course [19]. The regular teaching sessions held at MKT site appears to have been effective in maintaining skills and in some instances building on them. Our aim was to provide refresher teaching that is relevant to the audience and related to clinical exposure, which has been shown to have the greatest impact in previous studies [20,21]. The 6–8 week gap before the post-test was repeated was chosen with the intention of reducing the chance of confounding variables such as alternative training which had been cited as a problem to control in other studies [22-24]. However we note that this is at the cost of having a relatively short interval between participants gaining knowledge and being retested, when we are trying to assess how skills are retained over time. Future assessments could aim for a longer assessment interval however the MKT site suggests that reinforcement through teaching is likely to be required on a regular basis with the staff to maintain skills.

Confidence levels recorded by the participants (Table 4) increase with other scenarios as well as those tested indicating that the broad ABCDE approach equips staff with skills they feel they can apply to other acute presentations. However only by testing those other scenarios could we assess how well the participants apply those skills.

The reception by staff of such a training programme was more positive than expected; staff were very keen to attend the post-teaching assessment in addition to the teaching day, and take the opportunity to demonstrate the skills they had learned. A number of staff stayed after work or came in from home in order to undergo the assessments, beyond what was expected of them, suggesting a gap in skills that the staff themselves are cognisant of.

This project describes one strategy used to target the training of paramedical staff in a low resource setting. It highlights some of the challenges that can be encountered when trying to deliver a ‘standardized’ service in this setting. It demonstrates the need for ongoing practical training programmes alongside existing teaching courses and indicates that routine “on- the-job” training is not sufficient. Also importantly it emphasises the importance not only of training, but also of measurement of baseline skills to understand the *quality* of care being delivered when support staff such as the doctors are not on site. As pointed out by Scott et al [25], until recently in low resource settings, the emphasis has largely been on improving access to health care but it is also vital that what is available is of good quality and this relies on measurement of quality, and on effective training of staff. One of the problems of delivering quality assured health care in a resource poor setting is that ‘best practice’ guidance is mostly aimed at western medical facilities and so assumes a level of training and facility not available in rural primary care settings [26]. There are additional barriers to be considered: Myanmar’s education has suffered decades of neglect; the challenge is to provide quality assured health care with the ‘available’ health workers, in a model that copes with the inevitable gaps in knowledge: particularly in basic anatomy, physiology and pharmacology; that are unlikely to be unique only to this setting. While significant improvements in mortality amongst pregnant women and in neonates [12,27] have been observed from these same clinics, discerning improvements in emergency patients in the inpatient setting will be more difficult due to the lower caseload and lower mortality in this patient group. Adaptation of the WHO Maternal near-miss mortality tool [28] which is a benchmark with standardized criteria (clinical, laboratory, and management) could potentially be applied to in-patients in rural areas.

### Limitations

There was no concurrent control group who were not exposed to the training however with one teaching session per week at the clinics, a lack of text books, and no internet access, contamination from other trainings is very unlikely. Not all 71 participants took part in the pre-test, post-test or 8-week retest, indeed only 24 passed through all tests, nevertheless as a pilot project it serves as a starting point to address competence in rural and remote areas.

No direct feedback of mistakes made during tests was provided but it is likely to have enhanced learning [29]; no documentation of real patient scenarios (already described as a powerful tool in the delivery rooms in this setting [15]) were observed but these can be developed before deployment of future training. Novel and tested methods such as videotaping health workers during scenarios both for assessment and feedback should be investigated [30] especially since this technology has become very accessible.

This pilot tool had the presence of UK doctors trained in advanced life support who have been educated in an OSCE [30] environment and have clinical experience with the ‘ABCDE’ scenario. This may in part have contributed to the success of the course, nevertheless the tool package has been provided (Additional file 1) and can be delivered by any doctor familiar with basic life support and willing to commit time to the pre-testing, training and follow-up. We hope that this project will encourage further research in measuring outcomes related to both knowledge and competence following participation in training for paramedical staff, and in particular encourage new strategies to improved training quality and professional development for paramedical workers in resource limited settings.

## Conclusion

In conclusion, the existing level of emergency skills in this group of paramedical workers was limited at baseline. However with a simple teaching programme that can be delivered onsite with minimal resources, we were able to demonstrate significant improvements in basic emergency skills and participant confidence. When assessing how well skills are retained following the course we demonstrated that teaching of this type is most beneficial when it is part of longer-term refresher training for maintenance of knowledge and confidence. In a clinical setting these assessments are carried out as a team and do not take the linear form that is used when teaching and assessing. The next step would be to assess the impact on clinical practice in mortality and near miss morbidity at the clinics.

## Additional file

Additional file 1:
**Emergencies Teaching User manual.**

## Abbreviations

MKT: Mawkerthai
MLA: Maela
WPA: Wangpha
SMRU: Shoklo Malaria Research Unit
ALSO®: Advanced life support in Obstetrics®
NGOs: Non-governmental organisations
