# Supplementary material for: A tool to improve competence in the management of emergency patients by rural clinic health workers: a pilot assessment on the Thai-Myanmar border
Source: Confl Health. 2015 Apr 13;9:11. doi: 10.1186/s13031-015-0041-x (PMC4395965; doi:10.1186/s13031-015-0041-x)
Supplement: Additional file 1: — Emergencies Teaching User manual. [file 13031_2015_41_MOESM1_ESM.docx]

**Emergencies Teaching User manual**

**Contents:**

1. Timeline
2. Baseline assessment
3. Assessment score sheet
4. Instructors and interpreting
5. Structure of teaching day – timetable
6. Teaching materials
7. Fortnightly teaching format (held at one site)
8. Background and confidence questionnaire

**1. Flow of assessments and teaching**

Table 1 Timeline of Assessments and teaching

Baseline assessments at all 3 sites (pre-teaching)

Teaching half-day delivered twice at each site (to allow for maximum coverage)

Open to all Medics and Nurses.

1-2 weeks

Post-teaching assessments

8 weeks

MKT only receives fortnightly refresher session

Follow up assessments

2. **Assessment scenario**

The following scenario was used for all 3 assessments for all participants.

This was chosen as it was not an unusual case to present to the clinic sites and was design for the participant to need to give simple interventions.

The scenario was provided written in English and with Karen and Burmese interpreting available for every participant.

***“A 45 year old female was admitted to IPD with Pneumonia yesterday. Her breathing is fast and shallow. On admission she had a fever and was coughing green sputum. This morning when you go to take her vital signs you find her clammy and unresponsive.”***

After being given the scenario, each participant was asked to talk through the actions they would carry out in the order they would do them, as if this was a real patient. The vital signs were given only if the participant requested them/ stated that they would carry out that specific observation (these are recorded on the assessment score sheet below under the middle column). If participants said they would call for help they were told that it was on its way but that they should carry-on as if on their own whilst they are waiting. There was no time limit.

**3. Assessment score sheet**

**Participant name___________________Site______________Date__________________ Pre|_| or Post|_|**

**AIRWAY /2**

| *Candidate assessment/ action* | *Information if asked* | *Score* | |
| --- | --- | --- | --- |
| Airway clear? Speaking? +/- airway maneuvers ** | No obstruction visible, slight snoring | 1 |  |
| Oxygen high flow (>10 L) ** |  | 1 |  |

**BREATHING /5**

| *Candidate assessment/ action* | *Information if asked* | *Score* | |
| --- | --- | --- | --- |
| **Respiratory Rate **** | 30 | 1 |  |
| Chest movements ?symmetry  Tracheal tug?  accessory muscle use?  cyanosis  Trachea central | Coarse crepitations at right lung base | /3 max |  |
| **O2 Saturations **** | 89% | 1 |  |

**CIRCULATION /8**

| *Candidate assessment/ action* | *Information if asked* | *Score* | |
| --- | --- | --- | --- |
| **Pulse **** | 120 | 1 |  |
| **Blood pressure /capillary refill (in child) **** | 80/40 | 1 |  |
| Check urine output/ fluid balance/catheter | Not recorded | 1 |  |
| IV line inserted |  | 1 |  |
| **Fluid bolus- correct fluid ****  **-correct amount **** |  | 1  1 |  |
| **Temperature **** | 38.9 C | 1 |  |
| Antibiotics correct |  | 1 |  |

**DISABILITY /4**

| *Candidate assessment/ action* | *Information if asked* | *Score* | |
| --- | --- | --- | --- |
| **Conscious level (AVPU) **** | Groans to pain | 1 |  |
| **Blood sugar/dextrose **** | Normal | 1 |  |
| Pupils (size and reaction to light) | Reactive and equal | 1 |  |
| Seizures/ meningism? +/- diazepam correct dose | No evidence of seizures or meningism | 1 |  |

**EXPOSURE/EVERYTHING ELSE /6**

| *Candidate assessment/ action* | *Information if asked* | *Score* | |
| --- | --- | --- | --- |
| Head to toe/front and back exam - Include abdominal examination |  | 1 |  |
| **Calls for HELP at any time in scenario **** |  | 1 |  |
| **MS and HCT **** |  | 1 |  |
| **REASSESS after interventions **** | BP and PR the same after 1^st^ fluid bolus | 1 |  |
| Review notes and charts |  | 1 |  |
| Management and investigation plan |  | 1 |  |

Please circle:

| Total score | /25 | Pass | Fail |
| --- | --- | --- | --- |

**= Vital step. If missed out ANY then overall fail, (Though still want full score documented - Need >14 and all yellow boxes, to pass)

Abbreviations : AVPU= simple conscious level assessment where A=alert, V=voice, P=pain, U=unresponsive

BP= Blood pressure; HCT= Haematocrit; MS= malaria smear; PR=pulse rate

**Assessment score sheet explained:**

A tick was given in the right-hand column for each action correctly carried out (i.e. a tick for “Oxygen” if 10-15 litres was given). Also recorded the order of actions by writing a number for action starting at 1 for the first and so on.

After completion of the assessment the participant score was determined by adding up the number of ticks in the right hand column. In order to pass, each participant had to have a tick in each row where the font is in bold with asterix**.

The same score sheet, scenario and format was used for all 3 assessments (baseline, Post-teaching, and follow up)

1. **Instructors and interpreting**

The teaching in our case was carried out for 3 sites over 3 days with a morning and afternoon session at each site. The same format was used throughout.

3 instructors were involved all of whom had at least 4 months Emergency Department experience and had undergone a minimum of Advanced Life Support training in the UK.

There was one interpreter for each session who also had clinical experience working in the clinics as a medic. Full interpreting was given for all lectures. In the small group sessions, groups were split so that participants with good English were in each group and so could if needed interpret for others.

1. **Teaching day timetable**:

| Time | TIME required | SESSION | TRAINERS |
| --- | --- | --- | --- |
| 9.00 | 15 mins | Introduction |  |
| 9.15 | 15 mins | Case scenario |  |
| 9.30 | 30 mins | Lecture of A-E assessment revision |  |
| 10.00-10.30 | 30 MINS/ CATCHUP | BREAK |  |
| 10.30 | 15 mins | Demo scenario by instructors |  |
| 10.45 | 15 mins  15 mins  15 mins  15 mins | Practice scenarios (go around 4 rooms in groups of 4 and each participant takes turns to run scenario) |  |
| 11.45 | 30 MINS | Handover/ communication |  |
| 12.15 | 15 mins | SUMMARY/END SESSION 1 |  |
| 12.30 -13.00 | 30 MINS | LUNCH |  |
| 13.00 | 15 mins | Introduction |  |
| 13.15 | 15 mins | Case scenario |  |
| 13.30 | 30 mins | Lecture of A-E assessment revision |  |
| 14.00-14.30 | 30 MINS/ CATCHUP | BREAK |  |
| 14.30 | 15 mins | Demo scenario by instructors |  |
| 14.45 | 15 mins  15 mins  15 mins  15 mins | Practice scenarios (go around 4 rooms in groups of 4 and each participant takes turns to run scenario) |  |
| 15.45 | 30 MINS | Handover/ communication |  |
| 16.15 | 15 mins | SUMMARY/END SESSION 2 |  |

1. **Teaching materials**

Although PowerPoint lectures are presented here, the lectures were delivered using only a whiteboard.

Facilities required are listed below under each workshop heading.

- 1. **Teaching handout.**


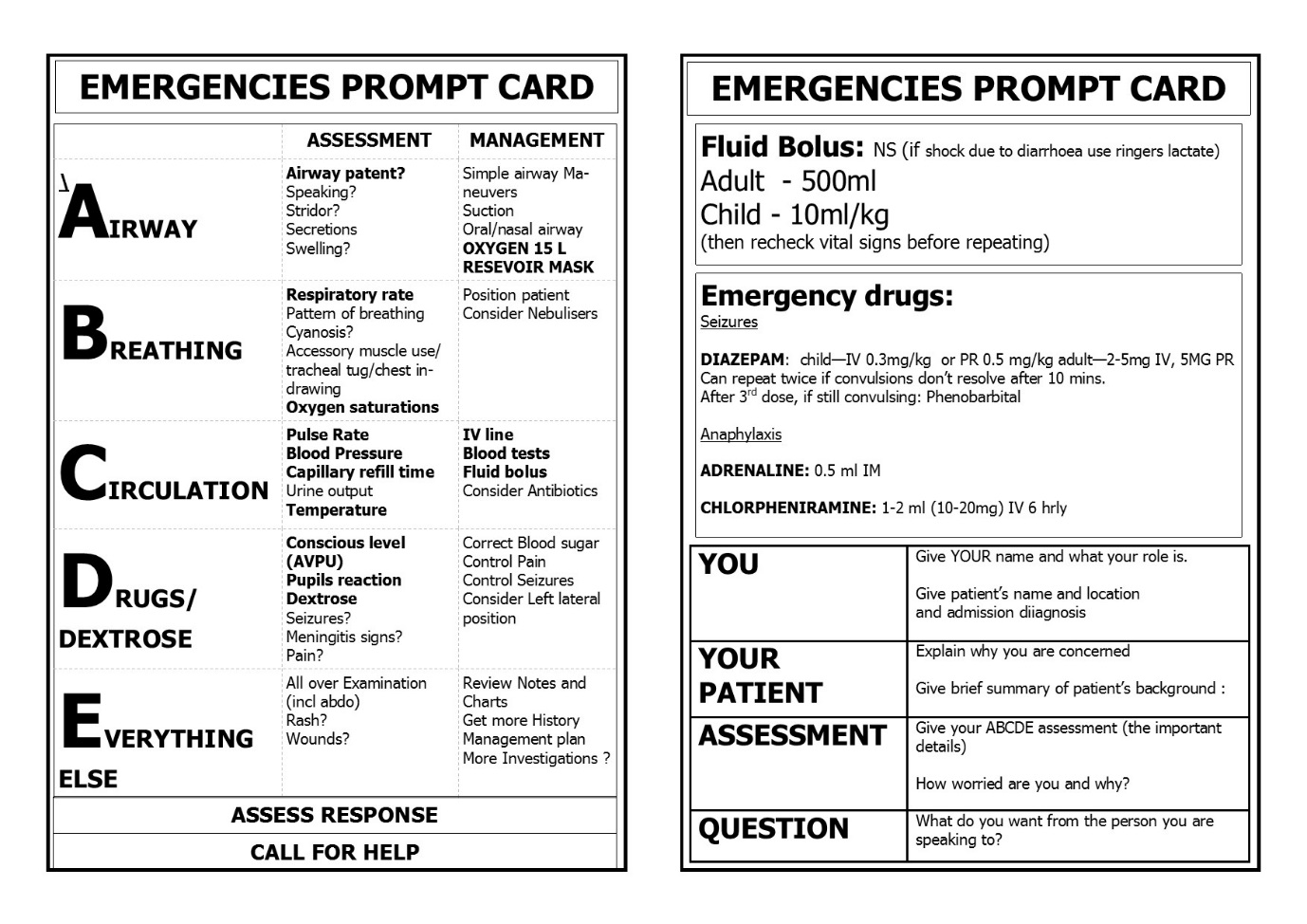


- 1. **Introduction + case scenario (lecture content)**

Real SMRU Case history:

- 34 year old female 28 weeks pregnant
- admitted with fever and abdominal pain
- Routine observations and blood tests taken
- Admitted to IPD

On admission:

- RR 26

- - O2 sats 96% - room air
  - Pulse 110/min
  - BP 120/55
  - Temp 38.0 C

The next day…

- RR 35

- - O2 sats 90% - room air
  - Pulse 140/min
  - BP 70/55
  - Temp 39.5 C
- Severe Pain
- Vomiting ++
- Not passing urine
- Drowsy

Outcome:

- Patient transferred to hospital but died later that day.

Could we improve?

|  | Admission | Day 2 | Day 3 |
| --- | --- | --- | --- |
| RR | 26 | 28 | 35 |
| O2 sats | 96% RA | 95% RA | 90% RA |
| Pulse | 110 | 115 | 140 |
| BP | 120/55 | 110/55 | 70/55 |
| Temp | 38.0 C | 37.5 | 39.5 |

Should we have intervened earlier? When?

Problems:

- Didn’t recognise importance of worsening vital signs
- Delay in response to deteriorating vital signs
- Slow to transfer / discuss with doctor

Summary:

- Important to know initial assessment and management of acutely unwell patients.
- Simple interventions and monitoring can make a big difference

-Oxygen

-Fluids

-Close Monitoring

- 1. **A-E assessment revision (lecture content)**

A= Airway

B= Breathing

C= Circulation

D=Disability and dextrose

E= Exposure/everything else

Airway Problems:

- Allergic reaction
- Reduced conscious level
- Foreign body, secretions
- Infection

Airway

| Look  Obstructions  Swelling  Secretions  See sawing  Colour | Listen  Can the patient speak?  Added noises:  Snoring  Crowing  Gurgling  Stridor | Feel  For airflow/ breaths at  the mouth and nose |
| --- | --- | --- |

Airway Management

- Ensure airway is open and maintained
- Simple airway manoeuvres
- Position
- Suction
- Consider using adjuncts

REASSESS

All Acutely Ill Patients :

Oxygen:

15L/min via a mask with reservoir bag

Breathing Problems:

- Infection
- Muscle weakness
- Pain
- Trauma
- Asthma/allergy

Breathing

| Look  Respiratory Rate  Pattern:  -Symmetrical?  -accessory muscles  Colour  O2 Saturations | Listen  full sentences?  Added sounds:  -Wheeze  -Crackles  SILENT CHEST?? | Feel  chest expansion  Symmetrical movement  Percussion(if trained)  Tracheal deviation |
| --- | --- | --- |

Breathing management

- Position
- Oxygen
- Bag valve mask
- Nebulisers

REASSESS!

Circulation problems:

- Sepsis
- Dehydration
- Blood loss
- Drugs

Circulation:

| **Look**  Colour  Haemorrhage  Urine output/ fluid balance  Any evidence of infection?  IV access? | **Listen**  Blood pressure  HYPOTENSION  IS A LATE SIGN | **Feel**  Pulse: character, rate  Temperature:  Central vs peripheral  Capillary refill time (CRT) |
| --- | --- | --- |

Circulation management:

- IV access
- Bloods +/- blood cultures
- **GIVE IV FLUID BOLUS:**  250-500mls over 5-10 minutes
- +/-urinary catheterisation

REASSESS!

Disability problems:

| **Primary**  **(inside the brain)** | **Secondary**  **(outside the brain)** |
| --- | --- |
| Brain injury  Epilepsy  Infection  Lesions | Hypoxia  Poor circulation/BP  Metabolic  alcohol/drugs |

Disability assessment:

- Conscious level:AVPU/GCS
  - Drowsiness/lethargy
  - New confusion/agitation
- Blood glucose
- Pupils
- Seizures
- Pain

| A | Alert |
| --- | --- |
| V | Responds to Voice |
| P | Responds to Pain |
| U | Unresponsive |

Disability management:

- Protect + manage the airway:?recovery position
- Correct blood glucose
- Control seizures
- Manage pain

REASSESS!

Exposure assessment:

Head to toe examination🡪 look for…

Rashes, abdominal wounds, surgical scars, abdominal distension, evidence of bleeding, calf swelling/redness

Completion of assessment

- Review notes:

- Observation chart

- Fluid balance charts

- Drug prescriptions

- Case notes

- Full history and systems review
- Monitoring Plan
- Document clearly
- Communicate with doctor? Consider transfer?
- REASSESS patient progress

If you are unsure, call for

**HELP !**

and reassess **A B C D E**

**6.4 Demo scenario by instructors**

ACUTE ABDOMEN ADULT:

A 25 year old man is admitted with severe Right Iliac Fossa pain. He has had the pain for 2 days but today it is much worse and he cannot walk or move without pain. He is normally fit and well. He is sweating and holding his abdomen.

- 1. **Practice scenarios**

Practice scenario 1

DEHYDRATED CHILD/HYPOVOLAEMIC SHOCK:

A 9 year old child is brought to the IPD by his father (carrying him). He tells you the child has been Un-well with fever and loose watery stools for 4 days at home. He is pale and drowsy.

Practice scenario 2

PREGNANT WOMAN SEPSIS:

A pregnant woman is brought to the IPD at night by her husband. She is 32 weeks pregnant and has been feeling generally unwell for the past 3 days with some abdominal/back pain. She thinks she may have a fever.

Practice scenario 3

ANAPHYLAXIS ADULT:

A 15 year old boy is rushed to the IPD from the local village. He developed worsening difficulty in breathing around 30 minutes ago and now has a blue colour to the lips. He was seen at

the IPD earlier in the day and treated with oral antibiotics for an abscess on his arm.

Practice scenario 4

SEIZURES CHILD:

A 3 year old child is brought to the IPD by her mother who says she suffered a seizure lasting 2 minutes that morning. She has been unwell with sore throat and cough for 1 day at home. Whilst you are talking to the mother the child’s eyes roll back and she begins to fit again.

- 1. **Handover/communication session**

| **YOU** | Give YOUR name and what your role is.    Give patient’s name and location and admission diagnosis |
| --- | --- |
| **YOUR**  **PATIENT** | Explain why you are concerned    Give brief summary of patient’s background : |
| **ASSESSMENT** | Give your ABCDE assessment (the important details)    How worried are you and why? |
| **QUESTION** | What do you want from the person you are speaking to? |

1. **Fortnightly teaching format (held at one site)**

This was carried out during the weekly allotted teaching time, once every two weeks, as a group. Each individual was asked to give the next action they would carry out and respond to information given, working through the ABCDE assessment as a group.

The following real patient scenarios were used:

Scenario1:

“16 year old girl is brought to the clinic by her family unconscious. They report she has had 3 seizures overnight and in between is drowsy and confused. She complained of a severe headache last night but has been otherwise well. She is 2 months post-partum.”

Halfway through your assessment she begins fitting again.

Scenario2:

“A 54 year old woman is brought the OPD by her son. She had been unwell will urinary frequency and dysuria for 3 days. This morning she went to the toilet and was found collapsed there after around 20 mins, she appeared to have hit her head. Her son says she has been drowsy and confused and unable to walk since. She normally works and is independent.” The patient has a dense left hemiparesis.

Scenario3:

“A 3 year old child is brought to the clinic by his mother who says he has had increasing difficulty breathing for the last 2 days. The child is not feeding and is breathing fast with chest in-drawing visible from the end of the bed.” The chest is clear on examination.

**8. Background and confidence questionnaire**

**Participant Information Questionnaire**

*** your answers here will not affect your role in any way but will help us to improve in the future****

**Name**………………………………………. **Age**……………… **Site**…………………………………..

**What is your role at SMRU? (circle)**

Medic Nurse Other………………….

**For how long have you worked for SMRU? (circle)**

Less than 1 year between 1 and 5 years more than 5 years

**When did you become qualified in your role (no longer a student)?**

Less than 1 year between 1 and 5 years more than 5 years

**Where have you learnt most of your skills for taking care of patients? (circle one)**

Training in Burma (Please describe/name) ………………………………………………………………………………………………….

Training in Thailand (Please describe/name) ………………………………………………………………………………………………

Training at SMRU (Please describe/name) …………………………………………………………………………………………………

Working in Burma (Please describe/name) ……………………………………………………………………………………………….

Working in Thailand (Please describe/name) …………………………………………………………………………………………….

Working at SMRU (Please describe/name) ………………………………………………………………………………………………..

**How confident do you feel managing patients with the following problems? (tick one for each )**

|  | **Very unsure** | **A bit unsure** | **A bit confident** | **Very confident** |
| --- | --- | --- | --- | --- |
| **Anaphylaxis** |  |  |  |  |
| **Acute abdominal pain** |  |  |  |  |
| **Acute Shortness of breath** |  |  |  |  |
| **Sepsis** |  |  |  |  |
| **The unwell pregnant woman** |  |  |  |  |
| **The unconscious patient** |  |  |  |  |

**Do you feel you need more training on emergencies? (circle one)**

Yes No

**How do you find your Training you have received from SMRU? (circle one)**

Poor average good excellent
